# Fucoidan-Based Gold Nanoparticles: Antioxidant and Anticancer Potential from *Turbinaria decurrens* and *Sargassum cinereum*

**DOI:** 10.3390/pharmaceutics17070826

**Published:** 2025-06-25

**Authors:** Ahmed S. El Newehy, Saly F. Gheda, Mona M. Ismail, Dara Aldisi, Mahmoud M. A. Abulmeaty, Mostafa E. Elshobary

**Affiliations:** 1 Botany and Microbiology Department, Faculty of Science, Tanta University, Tanta 31527, Egypt; anewehy@ksu.edu.sa (A.S.E.N.); salygheda@yahoo.com (S.F.G.); 2 National Institute of Oceanography and Fisheries, Cairo 4262110, Egypt; mona_es5@yahoo.com; 3 Department of Community Health Sciences, College of Applied Medical Sciences, King Saud University, Riyadh 11433, Saudi Arabia; daldisi@ksu.edu.sa (D.A.); mabulmeaty@ksu.edu.sa (M.M.A.A.); 4 Aquaculture Research, Alfred Wegener Institute (AWI), Helmholtz Centre for Polar and Marine Research, Am Handelshafen, 27570 Bremerhaven, Germany

**Keywords:** fucoidan, gold nanoparticles, anticancer activity, antioxidant properties, molecular docking, brown seaweed, cytotoxicity

## Abstract

**Background/Objectives:** Cancer remains one of the leading causes of mortality worldwide, while natural antioxidants have emerged as promising therapeutic agents in cancer treatment. Although fucoidan from brown algae shows anticancer potential, its efficacy is limited by bioavailability challenges, and the synergistic effects of combining it with gold nanoparticles remain unexplored. **Methods:** Fucoidan was extracted from *Sargassum cinereum* and *Turbinaria decurrens*. F-AuNPs were produced utilizing fucoidan as both a reducing and stabilizing agent. The nanoparticles were analyzed by UV-Vis spectroscopy, FTIR, TEM, XRD, DLS, TAG, and zeta potential evaluation. The antioxidant activity was evaluated by DPPH and FRAP tests. Cytotoxicity was determined against HepG2, THP-1, and BNL cells, utilizing MTT and SRB tests. Flow cytometry was utilized to assess the cell cycle, while molecular docking was carried out to examine binding to oncogenic proteins. **Results**: *T. decurrens* produced higher polysaccharides rich in fucoidan content (235.9 mg/g dry weight) and stated higher antioxidant activity (FRAP: 9.21 μg TE mg^−1^; DPPH: 4.48 μg TE mg^−1^) in comparison to *S. cinereum*. F-AuNPs showed potent cytotoxicity toward HepG2 cells, with IC_50_ values and cytotoxicity toward HepG2 cells, with IC_50_ values of 377.6 μg/mL for *S. cinereum* and 449.5 μg mL^−1^ for *T. decurrens*. Molecular docking revealed robust binding of fucoidan to COX-2 (−7.1 kcal mol^−1^) and TERT (−5.4 kcal mol^−1^). **Conclusions:** Fucoidan and F-AuNPs reveal remarkable antioxidant and anticancer properties. Nanoparticle formulation greatly improves bioactivity, underscoring its promise as a synergistic approach for cancer treatment by influencing oxidative stress and cancer-associated pathways.

## 1. Introduction

Cancer continues to pose a significant global health challenge, resulting in millions of fatalities annually. Despite advancements in chemotherapy, radiotherapy, and immunotherapy, these conventional treatments often lead to severe side effects, such as immunosuppression, organ toxicity, and multidrug resistance [1,2]. As a result, there is increasing interest in developing alternative therapeutic agents derived from natural sources that can selectively target cancer cells while minimizing toxicity. Marine-derived bioactive compounds, particularly polysaccharides, have emerged as promising candidates due to their antioxidant, anti-inflammatory, and anticancer properties [3,4,5].

Seaweeds are a particularly rich source of bioactive compounds, such as polyphenols, polysaccharides, and peptides, which have shown promise in different applications [6,7,8,9]. A review by Pereira and Valado [10] highlights the potential of seaweed-derived polysaccharides and phlorotannins in drug discovery, demonstrating their applications in anticancer, antimicrobial, and anti-inflammatory therapies. Studies have also demonstrated the antiproliferative effects of seaweed extracts. For instance, aqueous extracts from *Ecklonia maxima* and *Ulva rigida* have been shown to induce apoptosis in human liver cancer cells by promoting reactive oxygen species (ROS) production and disrupting mitochondrial membranes [11]. Another study reviewed the role of seaweed oligosaccharides in disease treatment, noting their therapeutic potential against cancer, diabetes, and obesity. Furthermore, a study on *Caulerpa lentillifera* emphasized its bioactive components, including phenolic compounds and polysaccharides, which exhibit antioxidant and anti-inflammatory properties beneficial for cancer therapy [12].

**Figure 1:**
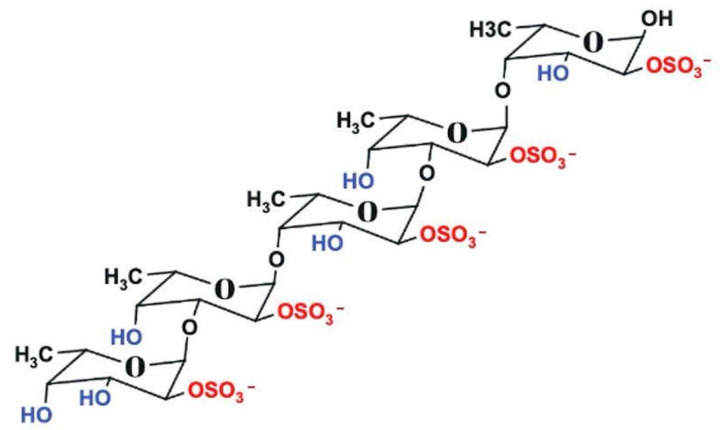
Schematic representation of the general structure of fucoidan, composed of sulfated α-L-fucose units with glycosidic linkages. Structure drawn using Marvin Sketch.

Among the promising natural compounds, fucoidan is a natural sulfated polysaccharide found in marine brown algae, characterized by a unique chemical structure with varying sulfation patterns, molecular weights, sugar compositions, and uronic acid content depending on their sources [13] (Figure 1). *Phaeophyceae* species are extensively studied for their bioactive composition, particularly their fucoidan content. These species are processed due to the bioavailability and bioactive properties of fucoidan. Because a large number of additional assets are deposited in the intracellular environment, *Sargassum* and *Turbinaria* algae have demonstrated anticancer and antioxidant capacities [14]. Hence, an anticancer agent and a potent natural radical scavenger and antioxidant can potentially be extracted from these algae. Best practices in cancer therapy include four critical factors: anticancer effects, chemical structure, antioxidant activity, and immune stimulation effects. Remarkably, fucoidan has demonstrated anticancer and antioxidant properties in several case studies [15]. The sulfated polysaccharides have the ability to interact with a wide variety of proteins, including cytokines, enzymes, and growth factors, thereby regulating cell-dependent events [16,17,18]. Additionally, fucoidan can reduce the concentration of reactive oxygen species and enhance the activity of various antioxidative enzymes, allowing for its potential use in drug design, particularly for cancer treatment and neuroprotection [19]. However, despite these promising effects, the clinical translation of fucoidan is hindered by challenges such as low bioavailability, poor stability, and limited cellular uptake. To overcome these limitations, researchers have explored various strategies to enhance fucoidan’s therapeutic potential [20]. Beyond fucoidan-based systems, Dong and Jiang fabricated ultrasmall platinum–iron nanozymes that catalytically decompose endogenous H_2_O_2_ to relieve tumor hypoxia and, under ultrasound, generate singlet oxygen to suppress tumor growth [21]. Likewise, Nie et al. engineered metal-organic-framework-coated MnO_2_ nanosheets capable of co-delivering doxorubicin and a surviving-targeting DNA zyme; stimulus-responsive degradation within the tumor milieu produced synergistic chemo gene therapy and chemo dynamic ROS generation [22].

One promising approach to improve fucoidan’s bioactivity is its combination with nanomaterials, particularly gold nanoparticles (AuNPs). AuNPs have gained significant attention in biomedicine due to their biocompatibility, ease of functionalization, and potential to enhance drug delivery [23,24]. When conjugated with fucoidan, gold nanoparticles may improve cellular uptake, increase bioavailability, and amplify anticancer effects by facilitating targeted delivery and controlled release [25]. Despite these advantages, the exact mechanisms through which fucoidan–gold nanoparticles (F-AuNPs) exert their therapeutic effects remain poorly understood, requiring further investigation. This study presents a novel comparative analysis of fucoidan extracted from *Sargassum cinereum* and *Turbinaria decurrens*, two ecologically and chemically distinct brown seaweeds. By comparing their influence on nanoparticle synthesis and bioactivity, this work adds new insights into seaweed-based nanomedicine.

Fucoidan-coated gold nanoparticles (F-AuNPs) were synthesized, characterized, and evaluated for antioxidant and cytotoxic activity. Molecular docking was used to explore interactions with cancer-related proteins. This integrative approach highlights the therapeutic potential of F-AuNPs as a novel anticancer strategy.

## 2. Materials and Methods

### 2.1. Collection and Identification of Macroalgae Samples

Two species of fresh brown seaweed were handpicked at a 1 m depth from submerged rocks from Hurghada City, Red Sea (27°17′13″ N, 33°46′21″ E), during summer 2023, as the most abundant species in that time. Algal samples were rinsed with seawater to remove sand; stored in plastic bags with seawater to prevent evaporation; and transported on ice to the Taxonomy and Biodiversity Laboratory, NIOF, Alexandria, for identification. The samples were cleaned of epiphytes and debris, rinsed with fresh water, and partially preserved in 5% formalin for taxonomic identification. The rest were shade-dried at 25–30 °C for 4–7 days, crushed into coarse powder, and stored in airtight bottles at −20 °C.

Taxonomic identification followed the classification systems of Taylor [26,27,28,29]. Species names were confirmed, using the AlgaeBase website [30], as *T. decurrens* Bory and *S. cinereum* J. Agardh.

### 2.2. Extraction of Polysaccharide Rich in Fucoidan

The polysaccharide rich in fucoidan extraction process, as described by Tako [31], involved suspending 10 g of powdered dry algae in 200 mL of 0.05 M HCl, followed by 2 h of magnetic stirring at room temperature. The mixture was then centrifuged at 4000 rpm for 20 min., and the resulting supernatant was filtered. The filtrate was neutralized using 0.5 M NaOH and subsequently precipitated by adding ethanol in a 2:1 volume ratio. The precipitated fucoidan was then subjected to freeze-drying, using a rotary evaporator. The yield of fucoidan was calculated as a percentage of the initial dry weight of the algae.

### 2.3. Characterization of Polysaccharide Rich in Fucoidan

#### 2.3.1. Fourier-Transform Infrared Spectroscopy (FTIR) Spectrum

The evidence of the presence of a polysaccharide rich in fucoidan was authorized by the Fourier-Transform Infrared Spectroscopy (FTIR) spectrum, using a Perkin Elmer 100 FTIR spectrophotometer (Waltham, MA, USA).

#### 2.3.2. X-Ray Diffraction (EDX)

The elemental composition of polysaccharides rich in fucoidan was estimated via energy-dispersive X-ray diffraction (EDX; JOEL JSM-IT200, Akishima, Tokyo, Japan).

### 2.4. Preparation of Fucoidan Solution

Two grams of the extracted polysaccharide rich in fucoidan powder were dissolved in 20 mL of dist. water and stirred until a uniform solution formed. The solution was then filtered using syringe filters (GD/X; 1.2 μm pore size; Whatman, UK) to eliminate any undissolved particles. The filtered solution was kept at 4 °C for later use.

### 2.5. Biosynthesis of Gold Nanoparticles (AuNPs)

A 10 mL aliquot of the fucoidan solution was added to 90 mL of 1 mM gold chloride solution (HAuCl_4_·3H_2_O) and stirred magnetically at room temperature. The color change during the reaction, indicating AuNP formation, was monitored using a UV-Vis spectrophotometer (Cintra 303). After formation, the AuNP solution was centrifugated at 10,000 rpm (Qiagen Sigma 4-15C) for 15 min, and the collected AuNPs were used for subsequent applications [32].

### 2.6. Characterization of Au-NPs

The formation of AuNPs was proven using a UV-Vis spectrophotometer (Shimadzu UV-2101/PC; Shimadzu Corporation, Kyoto, Japan) with a scanning range of 200–900 nm, encompassing ultraviolet, visible, and near-infrared regions. The Fourier-Transform Infrared Spectroscopy (FTIR) spectrum was obtained using a Perkin Elmer 1430 FTIR spectrophotometer. A pellet was prepared by blending 5 mg of the sample with 200 mg of FTIR-grade KBr. The FTIR spectra were recorded in the range of 400 to 4000 cm^−1^ [33].

X-ray diffraction (XRD) analysis was performed using an XRD-6000 (SHIMADZU) instrument with an incident beam (λ = 1.540598 Å) over a 2θ range of 5.1° to 80.1° to analyze the diffraction pattern of the AuNPs.

Transmission electron microscopy (TEM) imaging was established using Field Emission Transmission Electron Microscopy (NRC QUANTA FEG250). For observation, 10–20 μL of the prepared AuNP solution was dried on a copper grid. The zeta potential, particle size, and size dispersal were examined using dynamic light scattering (DLS) with a Zetasizer Nano ZN instrument at a fixed scattering angle of 173° and a constant temperature of 25 ± 0.1 °C to ensure the stability of the synthesized AuNPs.

The thermogravimetric analyses (TGAs) were performed using a TGA/DSC 1 STAR^e^ System (Mettler Toledo, Switzerland). Samples were heated from 25 to 800 °C, at a heating rate of 10 °C min^−1^.

### 2.7. In Vitro

#### 2.7.1. Antioxidant Activity

##### DPPH (2,2-Diphenyl-1-Picryl-Hydrazyl-Hydrate) Free Radical Scavenging Assay

The DPPH was conducted according to Boly et al. [34] with modifications from Elkhloly et al. [35]. A Trolox standard was used, and the AuNP solution was prepared at a concentration of 2.5 mg mL^−1^ in distilled water. In a 96-well plate, 100 μL of freshly prepared 0.1% DPPH dissolved in methanol was homogenized with 100 μL of the nanogold sample (*n* = 3) at different concentrations. The reaction mixture was incubated in the dark at 50–70 °C for 30 min. After incubation, the reduction in DPPH color intensity was measured at 540 nm.

##### Ferric-Reducing Antioxidant Power (FRAP) Assay

The FRAP assay followed the method outlined by Benzie and Strain [36], with samples prepared at a concentration of 12.5 mg mL^−1^ in distilled water. Trolox standards, ranging from 25 to 400 μg mL^−1^, were dissolved in methanol. The FRAP reagent was composed of 300 mM acetate buffer (pH 3.6), 10 mM TPTZ in 40 mM HCl, and 20 mM FeCl_3_, mixed in a 10:1:1 ratio. In a 96-well plate, 10 μL of the sample was combined with 190 μL of FRAP reagent (*n* = 3). The plate was then incubated in the dark at 25 °C for 30 min, and absorbance was recorded at 593 nm, using a FluoStar Omega microplate reader (BMG LABTECH GmbH, Ortenberg, Germany). Results were reported as μg Trolox equivalents per mg of sample.

#### 2.7.2. Cytotoxicity and Anticancer Effect

##### Cell Culture and General Conditions

Mouse liver cells (BNL), hepatocellular carcinoma cells (HepG2), and acute monocytic leukemia cells (THP1) were obtained commercially (Signa Aldrich, St. Louis, MO, USA) and cultured in the appropriate media. BNL and HepG2 cells were maintained in the DMEM medium, while THP1 cells were cultured in the RPMI medium. Both culture media were supplemented with 100 mg mL^−1^ streptomycin, 100 U mL^−1^ penicillin, and 10% heat-inactivated fetal bovine serum. The cells were incubated at 37 °C in a humidified atmosphere containing 5% CO_2_ [37,38,39].

##### SRB Assay for Normal BNL Cells

The SRB assay was performed to assess BNL cell viability. Cells (5 × 10^3^/well) were seeded in 96-well plates and incubated for 24 h, followed by treatment with varying concentrations of AuNPs. After treatment, cells were fixed with 10% TCA at 4 °C for 1 h, washed, and then stained with 0.4% SRB. Excess dye was removed with 1% acetic acid, and plates were air-dried overnight. Bound dye was solubilized in 10 mM TRIS buffer, and absorbance was measured at 540 nm. Results were expressed as a percentage of control [37,40].

##### MTT Assay for HepG2 Cells

HepG2 cell viability was evaluated using the MTT assay. Cells (5 × 10^3^/well) were seeded in 96-well plates and incubated for 24 h. They were then treated with 100 μL of medium containing different concentrations of AuNPs. After 48 h, the medium was removed and replaced with 20 μL of MTT solution (1 mg mL^−1^ in PBS). Following a 4 h incubation at 37 °C, the resulting formazan crystals were dissolved in 100 μL of DMSO, and absorbance was measured at 570 nm, using a FLUOstar Omega microplate reader (BMGLABTECH, Germany) [38,39].

##### WST-1 Assay for THP1 Cells

THP1 cell viability was assessed using the WST-1 assay (Abcam^®^ kit, ab155902, Abcam plc, Cambridge, UK). Cells (5 × 10^3^/well) were plated in 96-well plates and incubated for 24 h, followed by treatment with 50 μL of medium containing various concentrations of AuNPs. After another 24 h, 10 μL of WST-1 reagent was added, and absorbance was measured at 450 nm after 1 h, using an Infinite F50 microplate reader (TECAN, Männedorf, Switzerland) [41,42].

##### Cell Cycle Analysis by Flow Cytometry

HepG2 cells (1 × 10⁵) were seeded and treated with AuNPs for 48 h. After trypsinization, cells were washed twice with cold PBS (pH 7.4) and fixed in 2 mL of 60% ethanol at 4 °C for 1 h. The cells were then washed again and resuspended in PBS containing 50 μg mL^−1^ RNase A and 10 μg mL^−1^ propidium iodide (PI). Following a 20 min incubation at 37 °C in the dark, DNA content was analyzed using flow cytometry (ACEA Novocyte™; FL2 channel, λex/em 535/617 nm). Cell cycle distribution was determined from 12,000 events per sample using ACEA NovoExpress™ software version 1.4.1 (ACEA Biosciences Inc., San Diego, California (CA), USA) [43].

### 2.8. In Silico Study

#### 2.8.1. Target Prediction

SwissTargetPrediction ([http://www.swisstargetprediction.ch/] accessed on 15 January 2025) was used to identify potential biological targets of fucoidan in the Rat model. This web-based tool predicts likely macromolecular targets of small molecules by analyzing their 2D and 3D structural similarities to known ligands.

#### 2.8.2. Protein Preparation

The three-dimensional structures of the ALOX (P12527), COX-2 (P35355), H3R (Q9QYN8), TERT (Q673L6), and TYMS (UniProt ID: P45352) were obtained. The proteins were prepared for docking using the AutoDockTools 1.5.6 suite [44]. All non-protein atoms, including crystallographic waters and ligands, were eliminated. Polar hydrogen atoms were added, and the protein structures were cleaned by merging non-polar hydrogen atoms and assigning Gasteiger charges.

#### 2.8.3. Ligand Preparation

The three-dimensional structure of fucoidan (PubChem: 204) and Au gold was prepared using Avogadro 1.2.0 [45]. All ligand structures were prepared by adding hydrogen atoms, assigning partial charges (Gasteiger method), and performing energy minimization using the UFF force field.

#### 2.8.4. Binding Site Prediction

The potential binding site on the STAT3 protein was predicted using the CB-Dock web server [46], which employs a deep learning-based approach for identifying putative binding pockets and cavities.

#### 2.8.5. Molecular Docking

Molecular-docking simulations were achieved using AutoDock 4.2 [47], with the genetic algorithm. The AutoGrid 4 program was used to calculate grid maps for the predicted binding site with a grid box size of 20 Å × 20 Å × 20 Å and a grid spacing of 0.375 Å. The following docking parameters were used:

ga_pop_size: 500 (number of individuals in the population)

ga_num_evals: 2,500,000 (maximum number of energy evaluations)

ga_num_generations: 27,000 (maximum number of generations)

The docking calculations were performed with a maximum of 10 conformations for the ligand, and the results were ranked based on the estimated free energy of binding (predicted binding affinity).

### 2.9. Statistical Analysis

Statistical analysis was performed using SPSS software (version 23, IBM Corp., Armonk, NY, USA). All experiments included three independent replicates. One-way analysis of variance (ANOVA) was used to assess differences among experimental groups, followed by Tukey’s post hoc test for pairwise comparisons. A *p*-value of <0.05 was considered statistically significant.

## 3. Results

### 3.1. Identification of Macroalgae Samples

**Figure 2:**
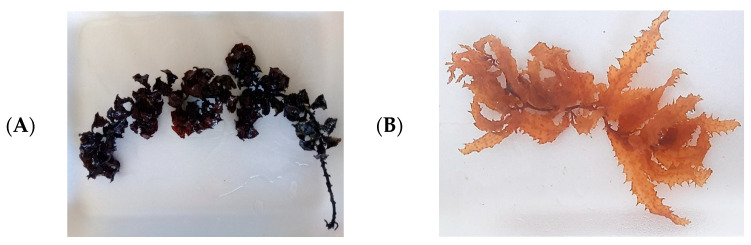
Photo of Morphological photographs of seaweed collected from the shores of the Red Sea: (**A**) *T. decurrens* Bory and (**B**) *S. cinereum* J. Agardh.

The collected seaweed species were identified and confirmed as *Sargassum cinereum* J. Agardh and *Turbinaria decurrens* Bory by Algae base database [48] (Figure 2)

### 3.2. Extraction of Polysaccharides Rich in Fucoidan

**Figure 3:**
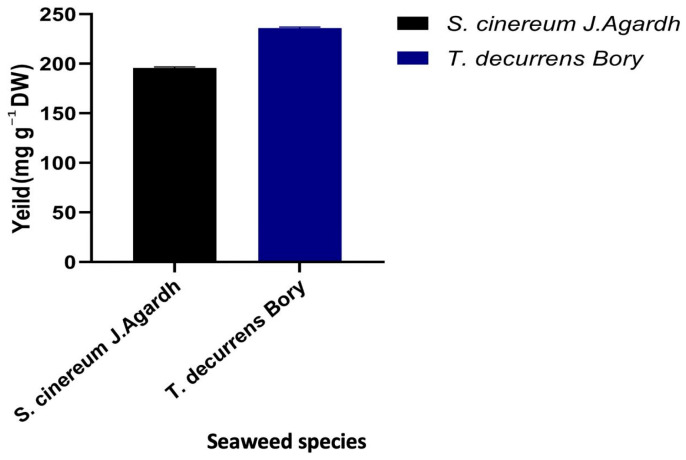
The yield of polysaccharides rich in fucoidan (mg g^−1^ dry weight) extracted from *S. cinereum* and *T. decurrens*.

The extraction of fucoidan from the two brown algal species, *T. decurrens* yields a higher quantity of polysaccharides rich in fucoidan (235.9 mg g^−1^ dry weight) than *S. cinereum* (195.8 mg g^−1^ DW), suggesting it as a more efficient source Figure 3.

### 3.3. Characterization of Polysaccharides Rich in Fucoidan

#### 3.3.1. Fucoidan–Gold Nanoparticle FTIR Spectroscopy Analysis

**Figure 4:**
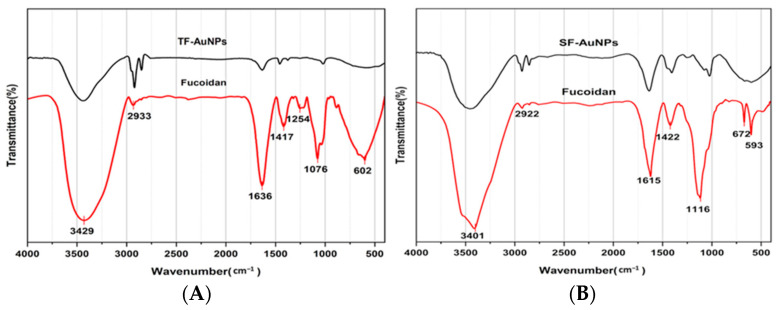
FTIR of polysaccharides rich in fucoidan extracted from (**A**) *T. decurrens* and (**B***) S. cinereum* and their nanoparticles.

FTIR spectra of fucoidan-rich polysaccharides from *T. decurrens* and *S. cinereum* exhibited broad O–H stretching bands at 3429 cm^−1^ and 3401 cm^−1^, respectively, confirming the presence of hydroxyl groups common in polysaccharides. C=O stretching bands were observed at 1636 cm^−1^ for *T. decurrens* and 1615 cm^−1^ for *S. cinereum*. A prominent S=O stretching band at 1254 cm^−1^ in *T. decurrens* indicates substantial sulfation, while *S. cinereum* showed a weaker sulfate band at 1116 cm^−1^, suggesting lower sulfation levels. A sharp peak at 1076 cm^−1^ in *T. decurrens* corresponds to C–O–C and C–O–S vibrations, indicative of glycosidic linkages and sulfated sugars, which appeared less distinct in *S. cinereum*. Low-frequency skeletal vibrations—linked to sugar ring deformation—were noted at 672 and 593 cm^−1^ in *S. cinereum*, and at 602 cm^−1^ in *T. decurrens* (Figure 4).

#### 3.3.2. EDX Analysis of Fucoidan Polysaccharides Rich in Fucoidan

**Figure 5:**
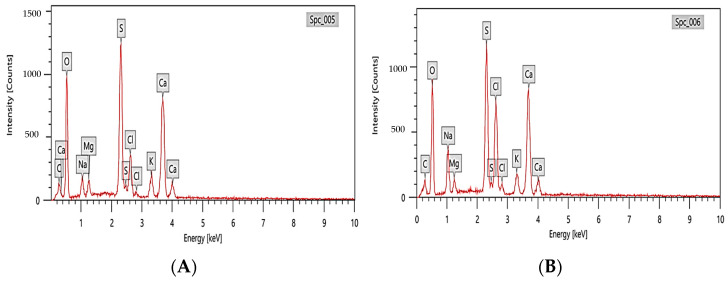
EDX data of polysaccharides rich in fucoidan extracted from (**A**) *T. decurrens* and (**B**) *S. cinereum*.

The results of energy-dispersive X-ray spectroscopy (EDX) of polysaccharides rich in fucoidan from *T. decurrens* and *S. cinereum*, shown in Figure 5, exhibited high levels of oxygen (O) and carbon (C), consistent with the carbohydrate backbone of polysaccharides. *T. decurrens* showed a higher oxygen content (48.59 ± 0.59%) compared to *S. cinereum* (42.45 ± 0.55%). Sulfur (S) was present in both samples, in which *T. decurrens* showed higher content (14.82 ± 0.17%) than *S. cinereum* (12.65 ± 0.16%), correlating with FTIR results showing more intense sulfate ester vibrations in the former. Other trace elements, such as sodium (Na), calcium (Ca), magnesium (Mg), potassium (K), and chlorine (Cl), were also detected in different amounts.

### 3.4. Characterization of Au-NPs

#### 3.4.1. Morphological Visual Observations

**Figure 6:**
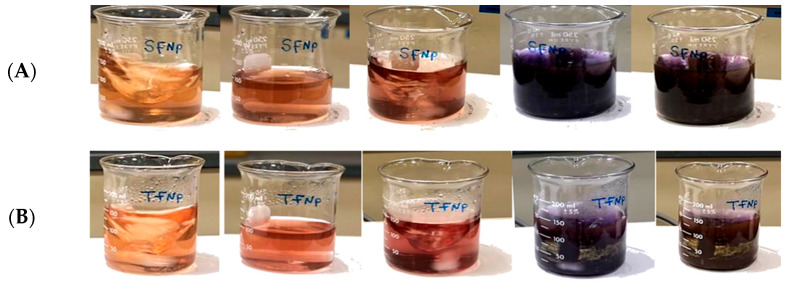
Formation of F-AuNPs using (**A**) *S. cinereum* and (**B**) *T. decurrens* fucoidan, showing a gradual color shift from yellow to reddish-brown over 2 h.

The gradually occurring color change suggested the formation of F-AuNPs over time in both *T. decurrens* and *S. cinereum* fucoidan extracts, showing a transition from yellow to reddish-brown, as shown in Figure 6A,B.

#### 3.4.2. UV–Vis Spectral Studies

**Figure 7:**
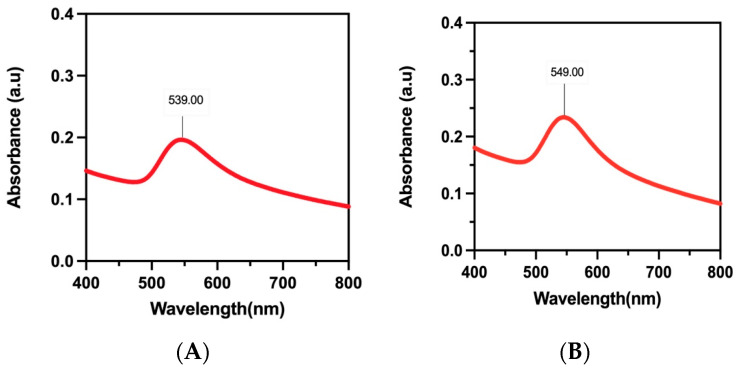
UV-Vis absorption spectrum curve analysis for F-AuNPs: (**A**) *T. decurrens* and (**B**) *S. cinereum*.

When gold nanoparticles are present, they exhibit a brown coloration due to surface plasmon resonance (SPR). This was demonstrated in studies of F-AuNPs derived from *T. decurrens* and *S. cinereum*, which showed characteristic SPR peaks at 549 nm and 539 nm, respectively (Figure 7A,B). These peaks fall within the visible-light region and are consistent with the known optical behavior of gold nanoparticles, further confirming their successful formation.

#### 3.4.3. Fucoidan–Gold Nanoparticle FTIR Spectroscopy Analysis

Upon synthesis of fucoidan-coated gold nanoparticles (F-AuNPs), FTIR spectra (Figure 4) revealed shifts and intensity changes in key functional group regions compared to fucoidan FTIR spectra. In *T. decurrens* F-AuNPs, O–H stretching appeared near 3400 cm^−1^, C–H vibrations at 2900–3000 cm^−1^, and distinct peaks at 1635 cm^−1^ (C=O) and 1420 cm^−1^ (COO^−^), confirming carboxylate groups. Sulfate group absorption was maintained at 1240 cm^−1^ (S=O) and 850 cm^−1^ (C–O–S). Similarly, *S. cinereum* F-AuNPs displayed bands at 3420 cm^−1^ (O–H), 2918 cm^−1^ (C–H), and 1640 and 1425 cm^−1^ (COO^−^), and sulfate peaks at 1235 and 845 cm^−1^. These findings confirm the successful functionalization of gold nanoparticles with fucoidan while retaining the structural integrity of the polysaccharide coating.

#### 3.4.4. X-Ray Diffraction Analysis of F-AuNPs

**Figure 8:**
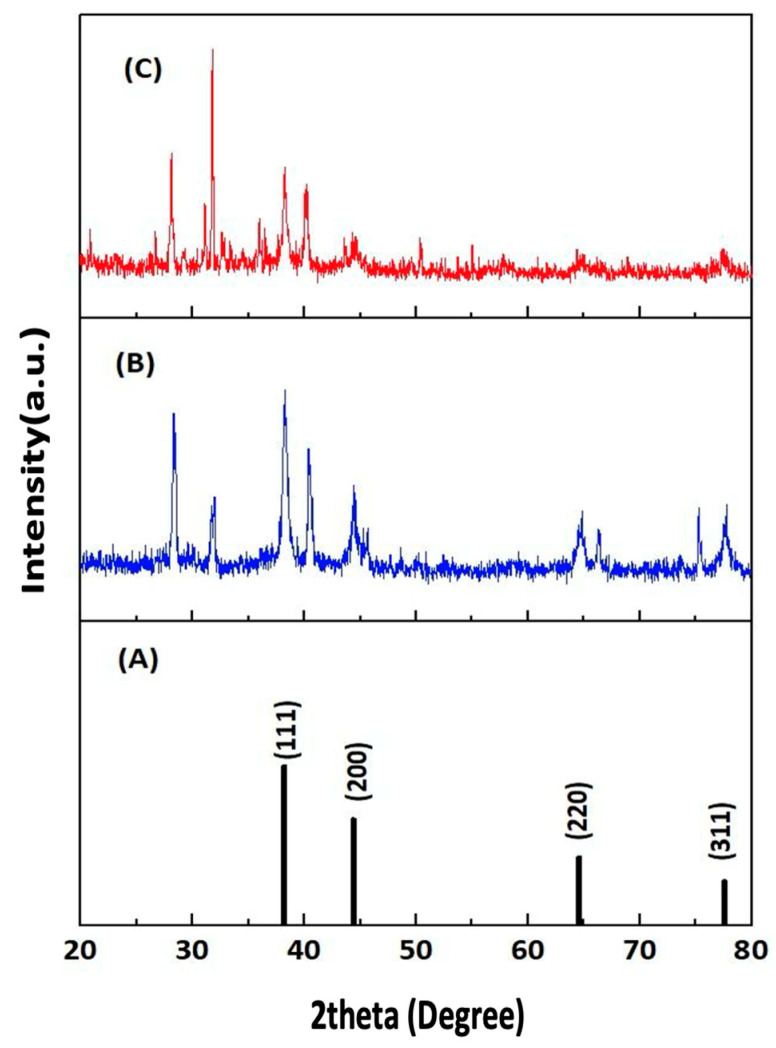
XRD pattern of Au (JCPDS file no. 04-0784) (**A**) and F-AuNPs of *T. decurrens* (**B**) and *S. cinereum* (**C**).

The crystalline nature of the synthesized fucoidan–gold nanoparticles (F-AuNPs) from *T. decurrens* and *S. cinereum* was confirmed through XRD analysis, as illustrated in Figure 8. The XRD pattern of *T. decurrens* F-AuNPs revealed distinct diffraction peaks at 2θ values of 13.87°, 28.44°, 31.84°, 38.36°, 40.53°, 44.53°, 64.87°, and 77.8° (Figure 8B). Among these, the peaks at 38.36°, 44.53°, 64.87°, and 77.8° can be indexed to the (111), (200), (220), and (311) Bragg’s reflections of the face-centered cubic (fcc) structure of metallic gold, respectively (Figure 8A).

Similarly, *S. cinereum* F-AuNPs exhibited characteristic peaks at 2θ values of 11.49°, 28.11°, 31.1°, 31.77°, 38.24°, 40.16°, 44.57°, 64.86°, and 77.76°. The peaks at 38.24°, 44.57°, 64.86°, and 77.76° correspond to the (111), (200), (220), and (311) crystallographic planes of gold nanoparticles (Figure 8C).

These XRD patterns confirm that both seaweed species yielded crystalline gold nanoparticles with a typical face-centered cubic structure, according to the Joint Committee on Powder Diffraction Standards (JCPDS file no. ICDD-PDF2, 04-0784, Newtown Square, PA, USA). The presence of additional peaks suggests a complex interaction between the fucoidan molecules and the gold nanoparticle surface, which could influence the stability and biological properties of the synthesized F-AuNPs.

#### 3.4.5. Transmission Electron Microscopy (TEM) Analysis of F-AuNPs

**Figure 9:**
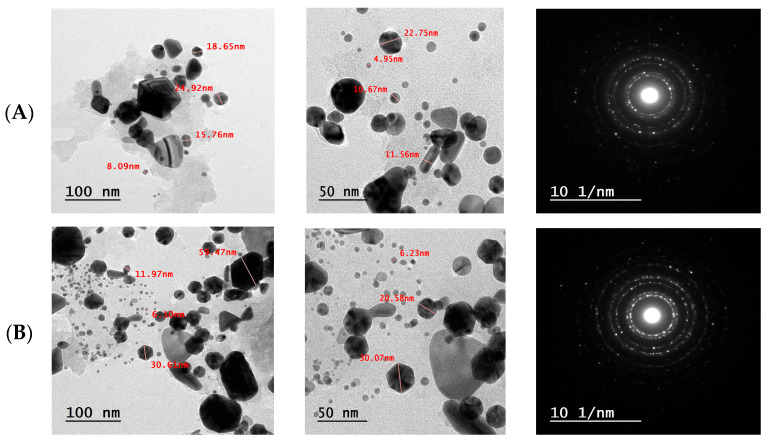
TEM images for F-AuNPs: (**A**) *T. decurrens* and (**B**) *S. cinereum*, and selected area electron diffraction (SAED) pattern.

The morphological characteristics and size distribution of the fucoidan-functionalized gold nanoparticles (F-AuNPs) synthesized from *T. decurrens* and *S. cinereum* were examined using TEM at different magnifications (Figure 9).

The (TEM) micrographs of *T. decurrens* F-AuNPs revealed predominantly spherical particles with some irregular shapes. Images taken at different scales (200 nm and 100 nm) demonstrated that the nanoparticles were well-dispersed, with some degree of aggregation. Particle size measurements indicated dimensions ranging from approximately 4.5 nm to 59.47 nm, showing a relatively uniform size distribution (Figure 9A).

Similarly, *S. cinereum* F-AuNPs exhibited mostly spherical morphology with occasional irregular shapes. The TEM images at various magnifications (200 nm and 100 nm) showed well-defined particles, with some clustering. The particle size analysis revealed dimensions ranging from 4.95 nm to 78.16 nm, indicating a slightly broader size distribution compared to *T. decurrens* F-AuNPs (Figure 9B).

In both cases, the TEM analysis confirms the successful synthesis of gold nanoparticles, with sizes ranging from very small (4–8 nm) to relatively large particles (>50 nm). This size distribution pattern suggests that while both seaweed species effectively facilitated the formation of gold nanoparticles, the growth and aggregation processes resulted in a heterogeneous size population. The presence of both smaller and larger particles might influence the overall properties and potential applications of these F-AuNPs. Interestingly, the selected area electron diffraction (SAED) pattern of AuNPs is also seen in Figure 8. The SAED pattern highlights variations in crystallinity and grain size among the samples. Polycrystalline characteristics are observed through the concentric rings. *T. decurrens* F-AuNPs exhibit more prominent and distinct rings, indicating smaller and uniform grains, while *S. cinereum* F-AuNPs display broader rings, suggesting larger or heterogeneous particle sizes. In the center-right corner, the SAED structure is displayed.

#### 3.4.6. Zeta Potential Analysis

**Figure 10:**
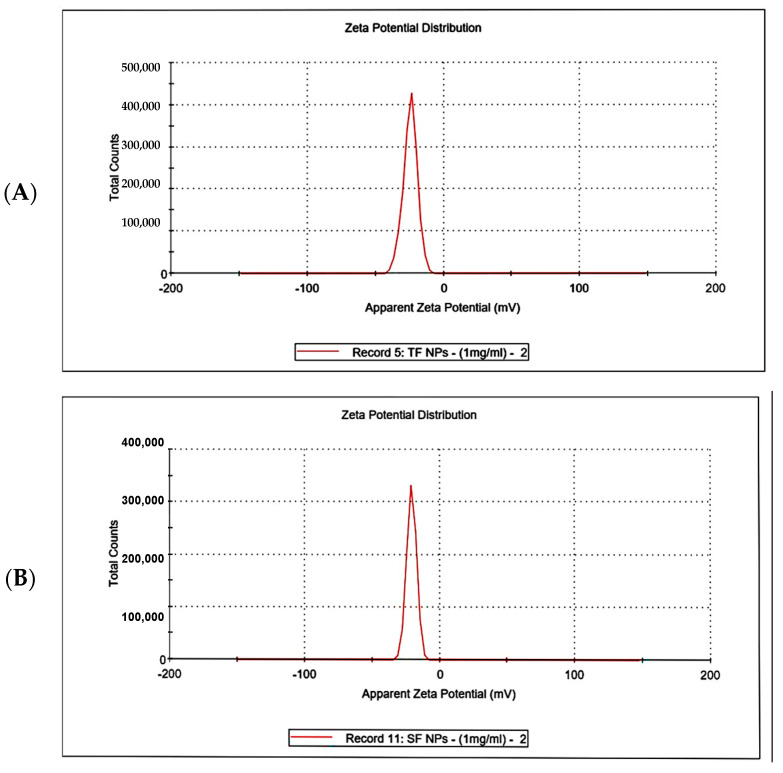
Zeta potential analysis of F-AuNPs for (**A**) *T. decurrens* and (**B**) *S. cinereum*.

Zeta potential analysis presented that the net charge of F-AuNPs from *T. decurrens* was −24.4 ± 5.23 mV, and the conductivity was 2.01 mS cm^−1^. In comparison, the net charge of F-AuNPs from *S. cinereum* was −20.7 ± 3.65 mV, and the conductivity was 1.53 mS cm^−1^. Moderate stability was expected for both F-AuNPs due to the presence of ionic species and electrostatic repulsion. *Turbinaria* exhibited less variability in zeta potential under the same conditions compared to *Sargassum* and showed slightly more stability over time. This may be attributable to the magnitude of its zeta potential, despite a higher standard deviation and, thus, variability in charge distribution, as shown in Figure 10A,B.

#### 3.4.7. Dynamic Light Scattering (DLS)

**Figure 11:**
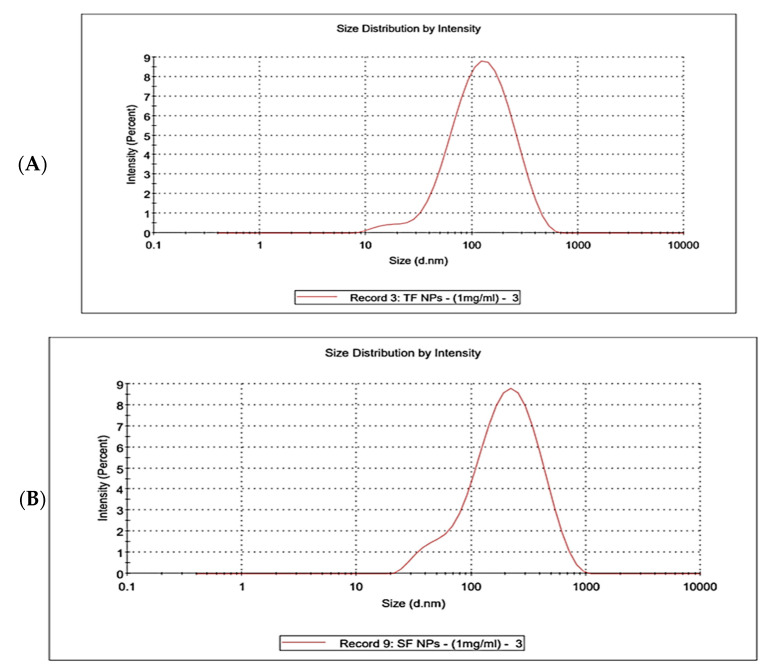
Dynamic light scattering (DLS) of particle size distribution of F-AuNPs: (**A**) *T. decurrens* and (**B**) *S. cinereum*.

Dynamic light scattering (DLS) analysis was performed to evaluate the hydrodynamic size distribution and colloidal stability of the synthesized fucoidan–gold nanoparticles prepared from *T. decurrens* and *S. cinereum*. The DLS results of *T. decurrens* and *S. cinereum* are represented in Figure 11A,B, Figure S1 and Figure S2. For *T. decurrens*, the zeta average was 98.5 ± 2.241 nm, with a Polydispersity Index (PdI) of 0.349 ± 0.031, indicating good homogeneity. For *S. cinereum*, the zeta average was 157.8 ± 5.552 nm, with a Polydispersity Index (PdI) of 0.390 ± 0.010.

#### 3.4.8. Thermogravimetric Analysis (TGA)

**Figure 12:**
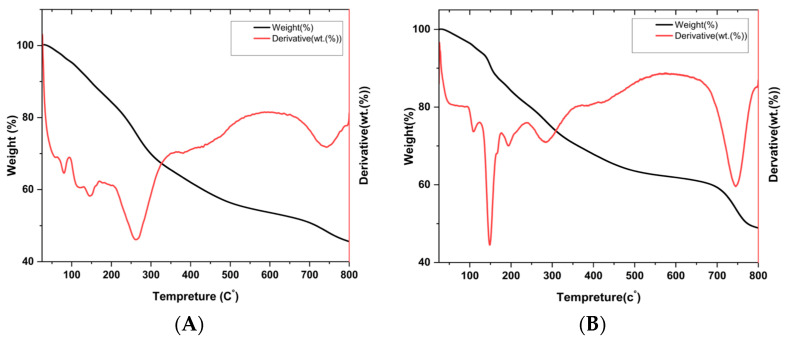
Thermogravimetric analysis of F-AuNPs of (**A**) *T. decurrens* and (**B**) *S. cinereum*.

Thermogravimetric analysis (TGA) was performed to assess the thermal stability and composition of fucoidan-capped gold nanoparticles synthesized using fucoidan extracted from two brown seaweed species: *T. decurrens* and *S. cinereum*. The TGA and DTG profiles (Figure 12A,B) provide insight into the thermal decomposition behavior of the organic fucoidan layer and the remaining inorganic core, confirming the successful capping of gold nanoparticles. In both samples, an initial weight loss of approximately 10–12% was observed between 50 and 150 °C, corresponding to the evaporation of surface-adsorbed water. The DTG curve of *T. decurrens* revealed a sharp peak between 250 and 350 °C, indicating a rapid and well-defined decomposition process, likely reflecting more uniform molecular architecture or lower branching of the *Turbinaria*-derived fucoidan. In contrast, *S. cinereum* exhibited a broader DTG profile over 200–500 °C, with multiple minor peaks, suggesting more complex degradation behavior. A secondary decomposition stage was noted in the 600–750 °C range, particularly in the *T. decurrens* sample. At 800 °C, the residual weight was approximately 45% for *Turbinaria* and 38% for *S. cinereum*, attributed to the thermally stable gold nanoparticles. The higher residual mass in TFNPS indicates a greater proportion of gold relative to the organic shell, while the lower residue in SFNPS suggests a higher organic content or thicker fucoidan coating. These findings confirm the formation of stable fucoidan–AuNP nanocomposites and highlight significant differences in their thermal degradation patterns based on the source of fucoidan. The range and sharpness of decomposition events, particularly those involving glycosidic bond cleavage, demonstrate how the structural characteristics of fucoidan influence the organic-to-inorganic ratio, thermal behavior, and possibly the functional properties of the nanoparticles.

### 3.5. Antioxidant Activity

#### 3.5.1. DPPH Free Radical Scavenging Assay

The DPPH free radical scavenging assay demonstrated that the fucoidan-based gold nanoparticles (F-AuNPs) exhibited significantly enhanced antioxidant activity compared to their corresponding crude fucoidan extracts across all tested concentrations (3.90–31.25 µg mL^−1^). For *Sargassum cinereum*, the F-AuNPs showed higher inhibition values, increasing from 10.82 ± 0.11% at 3.90 µg mL^−1^ to 80.49 ± 0.19% at 31.25 µg mL^−1^, compared to 7.92 ± 0.10% to 71.36 ± 0.21% for the fucoidan extract. Similarly, *Turbinaria decurrens* F-AuNPs achieved 12.05 ± 0.41% to 78.11 ± 0.31% inhibition, outperforming the native fucoidan (9.15 ± 0.25% to 73.09 ± 0.23%). Furthermore, the Trolox equivalent antioxidant capacity was higher in *S. cinereum* F-AuNPs (4.48 ± 0.42 µg TE mg^−1^) than in *T. decurrens* F-AuNPs (3.44 ± 0.11 µg TE mg^−1^) Table 1.

#### 3.5.2. Ferric-Reducing Antioxidant Power (FRAP) Assay

The ferric-reducing antioxidant power (FRAP) assay confirmed the superior redox potential of the fucoidan-based gold nanoparticles (F-AuNPs) over their parent polysaccharides throughout the tested range (25–400 µg mL^−1^). For *Sargassum cinereum*, nanoparticle treatment raised the inhibition from 0.07 ± 0.27 at 25 µg mL^−1^ to 1.12 ± 0.28 at 400 µg mL^−1^, whereas the crude fucoidan increased more modestly, from 0.02 ± 0.04 to 1.09 ± 0.12. A similar trend was observed for *Turbinaria decurrens*: its F-AuNPs rose from 0.07 ± 0.30 to 1.14 ± 0.19, surpassing the native fucoidan, which progressed from 0.04 ± 0.15 to 1.11 ± 0.01. When expressed as Trolox-equivalent antioxidant capacity, *T. decurrens* F-AuNPs delivered 9.21 ± 0.30 µg TE mg^−1^, marginally exceeding the 8.42 ± 0.27 µg TE mg^−1^ recorded for S. cinereum F-AuNPs. These data demonstrate that nano-engineering fucoidan markedly augments its electron-donating power, with T. decurrens-derived F-AuNPs displaying the strongest ferric-reducing ability among the materials evaluated (Table 2).

### 3.6. SRB Assay for Normal BNL Cells

**Figure 13:**
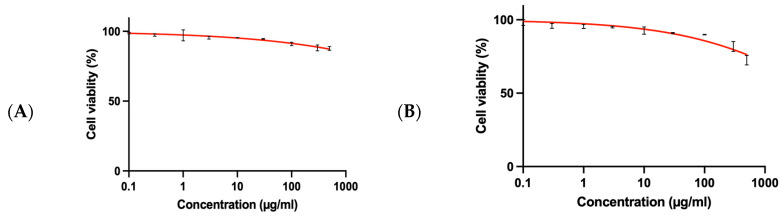
SRB assay for normal BNL cells: (**A**) *T. decurrens* and (**B**) *S. cinereum*.

The cytotoxicity of the fucoidan-derived gold nanoparticles (F-AuNPs) synthesized from *T. decurrens* and *S. cinereum* was assessed in normal murine hepatic cells (BNL) using the sulforhodamine B (SRB) assay. The dose–response curves (Figure 13A,B) indicate that cell viability exceeded 90% for all tested concentrations (0, 0.1, 1, 10, 100, and 1000 µg mL^−1^) and declined only marginally across the wider 0.1–700 µg mL^−1^ range. The extrapolated IC_50_ values were >1000 µg mL^−1^ for *T. decurrens* F-AuNPs and *S. cinereum* F-AuNPs, confirming negligible cytotoxicity toward normal liver cells and indicating a favorable safety margin for subsequent anticancer investigations. To quantify therapeutic selectivity, the selectivity index (SI) was calculated as IC_50_ (normal BNL)/IC_50_ (cancer HepG_2_). Because the IC_50_ in BNL cells lay beyond the highest concentration tested, these values represent lower-bound approximations SI > 2.2 for *T. decurrens* F-AuNPs and SI > 2.9 for *S. cinereum* F-AuNPs.

### 3.7. MTT Assay for HepG2 Cells and SRB Assay for THP1

**Figure 14:**
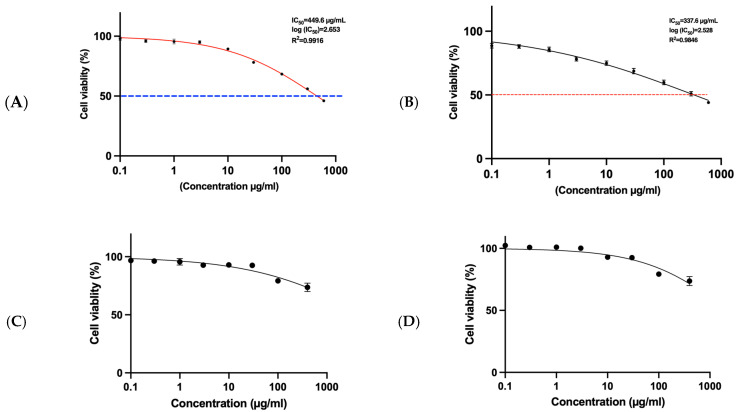
HepG1 (MTT) assay for (**A**) *T. decurrens* F-AuNPs, (**B**) *S. cinereum* F-AuNPs, (**C**) THP1 (SRB) assay of F-AuNPs *T. decurrens*, and (**D**) F-AuNPs *S. cinereum*.

The MTT assay is a widely employed colorimetric technique used to assess cellular metabolic activity, commonly interpreted as an indicator of cell viability, proliferation, and cytotoxic potential. In this study, fucoidan-mediated gold nanoparticles (F-AuNPs) synthesized from *Turbinaria decurrens* and *Sargassum cinereum* were evaluated for their cytotoxic effects on hepatocellular carcinoma cells (HepG2), using the MTT assay, and on acute monocytic leukemia cells (THP1), using the sulforhodamine B (SRB) assay. The cytotoxicity profile revealed that F-AuNPs derived from T. decurrens and S. cinereum exhibited significant activity against HepG2 cells, with IC_50_ values of 449.5 µg mL^−1^ and 337.6 µg mL^−1^, respectively (Figure 14A,B). When combined with the IC_50_ values in BNL cells > 1000 5 µg mL^−1^ for *T. decurrens* and *S. cinereum*, the corresponding SI values confirm ≥ 2-fold selectivity toward HepG_2_ over normal hepatocytes, with the *S. cinereum* nanoparticles showing the greater SI. These findings suggest that *S. cinereum*-derived F-AuNPs demonstrate relatively higher anticancer efficacy compared to those from T. decurrens and may hold therapeutic promise as potential nanomedicine agents for hepatocellular carcinoma.

Conversely, the same F-AuNPs exhibited negligible cytotoxic effects against THP1 cells, as evidenced by higher IC_50_ values (Figure 14C,D), indicating minimal toxicity within the tested concentration range. These results underscore the selective cytotoxicity of the formulations and support their continued investigation in terms of safety profiling and targeted anticancer applications.

### 3.8. Cell Cycle Analysis by Flow Cytometry

Flow cytometry analysis evaluated the effect of F-AuNPs synthesized from *T. decurrens* and *S. cinereum* on the cell cycle distribution of hepatocellular carcinoma cells (HepG2) in different phases of the cell cycle (G0/G1, S, and G2/M) to assess potential antiproliferative effects. The control group’s cell distribution was as follows: G0/G1 accounted for (54.90%) of the total, S phase (17.58%), and G2/M for about (27.52%). In contrast, cells exposed to Formulation T exhibited a significant increase in the G0/G1 phase (67.08%), alongside a notable decrease in the S phase (10.06%), G2/M about (22.69%), and a minor Sub-G1 population, indicating minimal apoptotic activity. These results indicate that Formulation T induces G0/G1 phase arrest, highlighting its antiproliferative effect by halting the progression of cells into the S phase.

**Figure 15:**
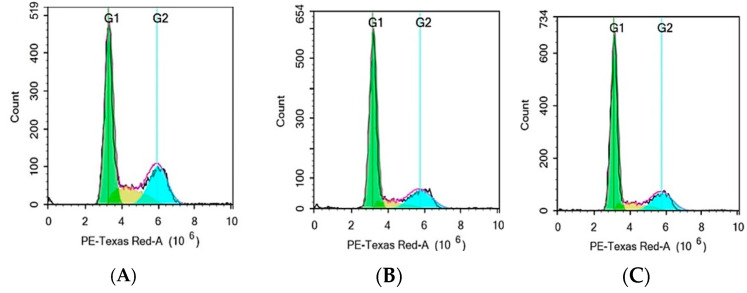
Flow cytometry analysis: (**A**) control, (**B**) *T. decurrens*, and (**C**) *S. cinereum*. Green region: Represents the G1 phase, typically indicating cells with a single set of DNA (2N); Yellow region: Likely corresponds to the S phase, where DNA synthesis occurs (between 2N and 4N content); Blue region: Represents the G2/M phase, where cells have duplicated DNA content (4N).

**Figure 16:**
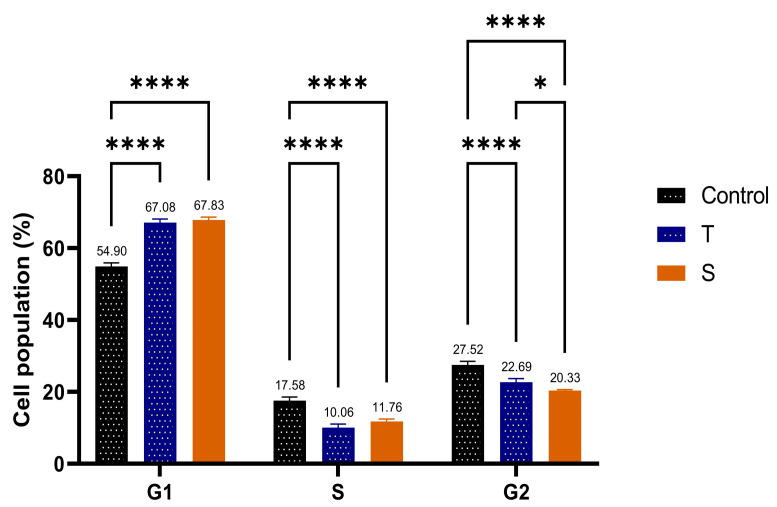
Comparison of cell cycle distribution across different phases (G1, S, and G2/M) in HepG2 cells treated with Formulations T and S, compared to the control. *, indicating statistical significance at *p* < 0.05 and ****, indicating statistical significance at *p* < 0.0001.

For Formulation S, a comparable effect was observed, resulting in an increased G0/G1-phase population (67.89%) and a decreased S-phase population (11.77%), with G2/M comprising about 20.23%, along with a minor Sub-G1 population. The results indicate that Formulation S, similar to Formulation T, produces G0/G1 phase arrest, thereby inhibiting the transition of cells into the S phase. The data collectively indicate that both formulations possess antiproliferative effects via modifying the normal cell cycle distribution in HepG2 cells, mainly by affecting the G0/G1 phase (Figure 15A–C and Figure 16).

### 3.9. Molecular Docking

**Figure 17:**
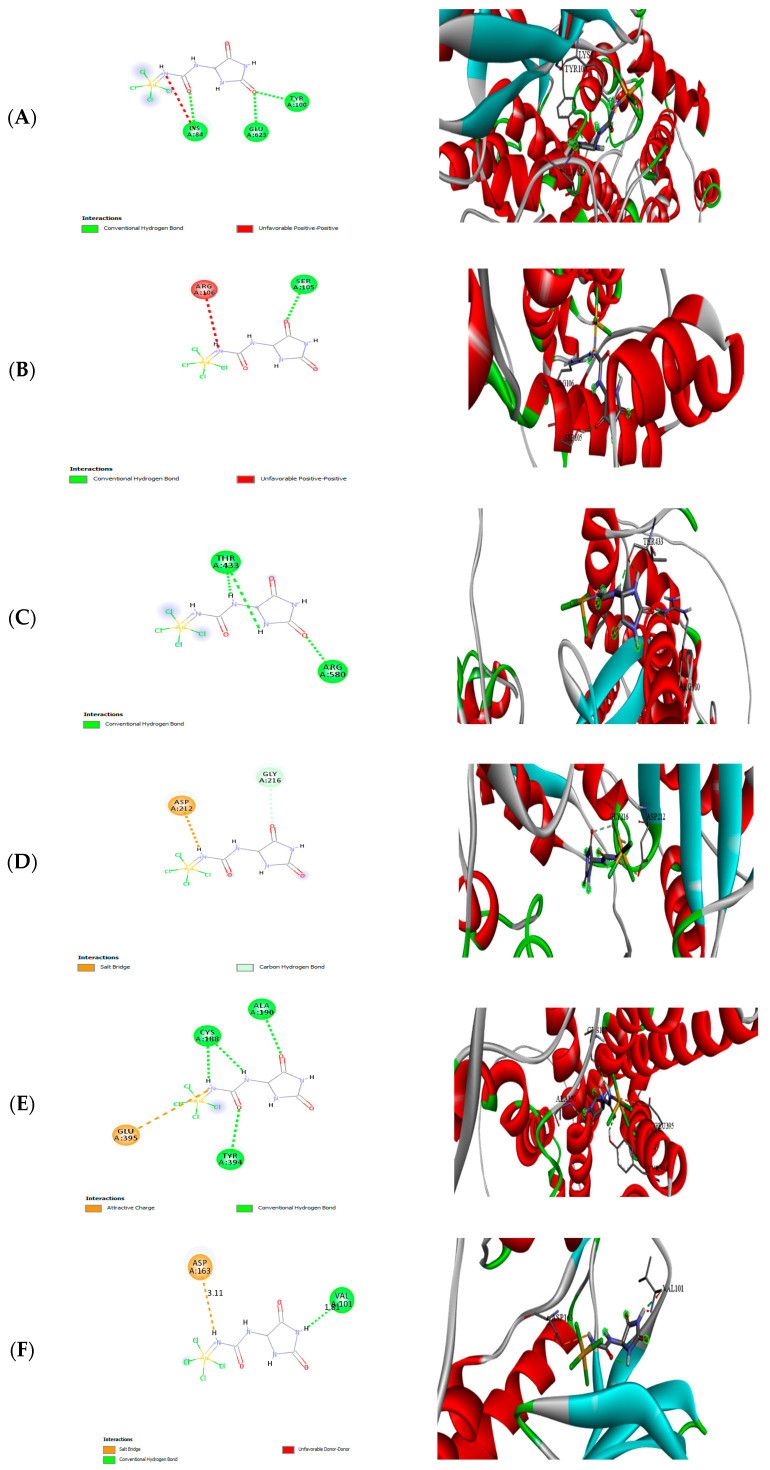
Molecular-docking analysis: (**A**) (2D and 3D) binding of F-Au NAPs with the active sites of arachidonate 5-lipoxygenase; (**B**) (2D and 3D) binding of F-Au NAPs with the active sites of transcriptase; (**C**) (2D and 3D) binding of F-Au NAPs with the active sites of telomerase reverse transcriptase; (**D**) (2D and 3D) binding of F-Au NAPs with the active sites of thymidylate synthase; (**E**) (2D and 3D) binding of F-Au NAPs with the active sites of histamine H3 receptor; and (**F**) (2D and 3D) binding of F-Au NAPs with the active sites of Cyclin-Dependent Kinases. Color key (2D interaction diagrams): Green dashed lines represent conventional hydrogen bonds; red dashed lines indicate unfavorable donor–donor interactions; orange dashed lines represent salt bridges or attractive charge interactions. Residues are color-coded based on interaction type (e.g., green for hydrogen bonding partners, red for unfavorable interactions, and orange for electrostatic interactions). Color key (3D structural views): Protein α-helices are shown in red, β-sheets and coils in cyan and grey, respectively. Interacting amino acid residues are rendered as green sticks, while ligands are depicted as multicolor ball-and-stick models to highlight atomic diversity.

Molecular docking was conducted to explore the interaction between F-AuNPs with target proteins to predict inhibition, with the results presented in Figure 17A–F and Table 3.

The molecular-docking study illustrated interactions between fucoidan–gold nanoparticles (F-AuNPs) and target proteins, as illustrated in Figure 17. The F-AuNPs exhibited notable binding affinities for multiple target proteins. Arachidonate 5-lipoxygenase (ALOX5) exhibited a binding energy of (−4.07 kcal mol^−1^). The dioxygenase is crucial in leukotriene biosynthesis and lipid peroxidation, significantly affecting inflammation and cell death mechanisms, such as apoptosis, pyroptosis, and ferroptosis.

These results suggest that F-AuNPs may inhibit oxidative stress and inflammation by targeting ALOX5 and COX-2, thereby reducing lipid peroxidation and pro-inflammatory mediators. This mechanism correlates with the observed antioxidant activity in DPPH and FRAP assays, highlighting the enhanced free radical scavenging capabilities of F-AuNPs derived from *S. cinereum* and *T. decurrens*. Additionally, the interaction with TERT suggests the potential of F-AuNPs to inhibit telomerase activity, a critical factor in cancer cell proliferation and immortality, which is supported by the observed G0/G1 cell cycle arrest in HepG2 cells. Furthermore, interactions with TYMS and CDKs indicate an inhibition of DNA synthesis and cell cycle progression, reinforcing the antiproliferative effects noted in the cytotoxicity assays. The strong binding affinity to H3R suggests an immunomodulatory role, which may complement the anticancer effects of F-AuNPs. The conjugation of fucoidan with gold nanoparticles likely enhances cellular uptake and stability, as demonstrated by lower IC_50_ values and superior antioxidant activity compared to fucoidan alone. These findings bridge the in silico and in vitro analyses, providing a robust mechanistic foundation for the therapeutic potential of F-AuNPs in oxidative stress-related diseases and cancer.

## 4. Discussion

The variations in fucoidan yield may be attributed to variations in species-specific cell-wall compositions, polysaccharide structures, or environmental factors influencing algal growth [49]. Previous studies have reported that extraction conditions, including pH, temperature, and solvent ratio, significantly impact the yield and purity of fucoidan [50].

*T. decurrens* yielding 23.8% of its dry weight appears exceptionally high, surpassing *S. cinereum* yields. This highlights its strong potential as a commercially viable fucoidan source, especially when compared to *S. cinereum*, and suggests species-specific advantages that merit further exploration. A comparable study on *Turbinaria* sp. reported the highest fucoidan yield among three tested brown seaweeds: *Turbinaria* sp.,* Sargassum* sp., and *Padina* sp. [51]. This highlights the superior fucoidan-producing potential of *Turbinaria* compared to other common brown algae.

FTIR analysis of fucoidan and F-AuNPs from *T. decurrens* and *S. cinereum* revealed prominent O–H stretching bands from 3000 to 3500 cm^−1^, characteristic of hydroxyl groups in polysaccharides. Peaks near 1636–1622 cm^−1^ indicate C=O stretching, commonly associated with uronic acids. Notably, *T. decurrens* showed a stronger sulfate ester band at 1215.43 cm^−1^ (S=O), while *S. cinereum* displayed a weaker sulfate signal around 1153.26 cm^−1^, indicating a higher degree of sulfation in *T. decurrens*. Upon synthesizing fucoidan-coated gold nanoparticles (F-AuNPs), FTIR spectra showed significant shifts and intensity changes [52,53].

These findings align with Palanisamy et al. [54], who reported similar spectral features across multiple seaweed species. Their study identified typical fucoidan peaks, including O–H vibrations (3367–3381 cm^−1^), C–H stretching in pyranoid rings (2930–2940 cm^−1^), carboxylate bands (1616–1629 cm^−1^), and sulfate groups (1220–1230 cm^−1^). Additional bands observed include C–H bending (1413–1421 cm^−1^), hemiacetal stretches (1024–1028 cm^−1^), and C–O–S bending (822–849 cm^−1^), all supporting the structural consistency of fucoidans across brown seaweed species such as *Sargassum wightii* [55], *S. polycystum*, [54], and *Turbinaria ornata* [56]. Additionally, the signals observed at 1466 and 1388 cm^−1^ may indicate the presence of uronic acid residues in the fucoidan polymer from *T. decurrens* [57]. This is supported by Khan et al., [58] who reported that fucoidan exhibits characteristic FTIR peaks at 845 cm^−1^ and within the 1159–1260 cm^−1^ range, corresponding to S=O asymmetric stretching and C–O–S stretching vibrations of sulfate groups, respectively.

The X-ray diffraction (XRD) technique was employed to investigate the crystalline structure of the extracted polysaccharides and to gain insight into polymer-related properties, such as molecular arrangement, flexibility, swelling behavior, and solubility. XRD patterns for fucoidan were recorded over a 2θ range of 5° to 80°. In the case of fucoidan-coated gold nanoparticles (F-AuNPs), corresponding to patterns B and C, the characteristic diffraction peaks of gold remain clearly observable. This confirms the successful formation of crystalline gold cores within the biogenic nanocomposites. Notably, in both samples, the (111) reflection appears as the most intense peak, indicating a preferred crystalline orientation along this plane—a typical feature of spherical or quasi-spherical AuNPs synthesized via biological methods [52]. However, the broader and more diffuse low-intensity peaks in both samples, especially in *S. cinereum*, suggest subtle differences in the amorphous structure of the polysaccharide coating, reflecting species-specific variations in fucoidan architecture.

In addition to the sharp gold-specific peaks, patterns B and C exhibit broader, low-intensity diffraction features, which are absent in the standard gold pattern (pattern A). These are attributed to the amorphous or semi-crystalline structure of the fucoidan-rich polysaccharide matrix derived from Turbinaria decurrens and Sargassum cinereum. Such features are consistent with prior reports on biologically synthesized or biomolecule-functionalized metallic nanoparticles, where the organic capping agents contribute to diffuse diffraction signals or additional low-intensity peaks, reflecting their disordered molecular structures [59]. This variation in crystallinity reinforces that different fucoidan structures influence nanoparticle properties differently.

Gold nanoparticles typically exhibit surface plasmon resonance (SPR) absorption in the 500–550 nm range, producing a characteristic brown coloration. In this study, F-AuNPs synthesized from *T. decurrens* and *S. cinereum* showed distinct SPR peaks at 549 nm and 539 nm, respectively, confirming nanoparticle formation, due to the unique characteristics of the two fucoidan types. These SPR signals are influenced by particle size, shape, composition, and interparticle distance [60,61,62].

FTIR spectra of both F-AuNP samples further confirmed successful functionalization with fucoidan, preserving key sulfated polysaccharide features. *S. cinereum* and *T. decurrens* F-AuNPs showed strong absorption bands, indicating S=O, C=O, and O–H groups, consistent with findings by El-Sheekh et al. [50] and Lee et al. [63]. Similarly, *S. cinereum* F-AuNPs displayed comparable spectral profiles, albeit with variations in intensity, supporting the effective coating of gold nanoparticles with fucoidan.

The size of nanogold particles varies depending on synthesis methods, stabilizers, and biological sources. Transmission electron microscopy (TEM) analysis of *T. decurrens* F-AuNPs revealed predominantly spherical particles with some irregular shapes, ranging from 4.5 nm to 59.47 nm, while *S. cinereum* F-AuNPs exhibited a similar morphology with a size range of 4.95 nm to 78.16 nm. The slightly larger and broader size distribution in *S. cinereum* may stem from its fucoidan’s more heterogeneous or less sulfated structure, which can affect nucleation and growth of nanoparticles. Comparable studies on gold nanoparticles synthesized from *Rhaphidophora aurea* extracts reported spherical particles with diameters of 10 nm to 35 nm [64]. Similarly, Au nanoparticles used in catalysis displayed sizes influenced by dopant modifications, with TEM confirming the changes [65,66,67]. The majority of nanoparticles produced through this green synthesis method are distinctly separated and possess diameters ranging from 10 to 50 nm, with a middling particle size of 39 nm [58]. These findings indicate that nanogold particle sizes fall within the observed range of *T. decurrens* and *S. cinereum* F-AuNPs, confirming their nanoscale properties. Further optimization in synthesis methods can influence size distribution and morphology for targeted applications.

SAED analysis shows crystallinity differences across samples. The semi-spherical NPs form through ultrasonic cavitation, where collapsing bubbles create extreme conditions (5000 K, 1000 atm) that control particle size [59]. SAED patterns confirm that the biosynthesized gold nanoparticles have single-crystalline, face-centered cubic structures [60].

The selected area electron diffraction (SAED) pattern confirmed the crystalline nature of the biosynthesized gold nanoparticles, with diffraction spots indexed to a face-centered cubic (FCC) structure, indicating the formation of well-defined, individual crystalline particles [68].

Zeta potential analysis revealed that F-AuNPs synthesized from *T. decurrens* and *S. cinereum* carried net surface charges of –24.4 ± 5.23 mV and –20.7 ± 3.65 mV, respectively, with conductivities of 2.01 and 1.53 mS cm^−1^. The more negative zeta potential in *T. decurrens*-derived F-AuNPs suggests improved colloidal stability, likely due to higher sulfate and carboxylate content, enhancing electrostatic repulsion between particles. These moderately negative values suggest incipient colloidal stability, consistent with the presence of negatively charged functional groups, such as carboxylates, on the nanoparticle surface [69,70,71]. The observed surface charge likely results from the adsorption of anionic species, including OH^−^ or citrate ions, during synthesis [69,70]. The high negative zeta-potential values confirm the presence of negatively charged carboxylate groups coating the nanoparticles. Suspended particles experience repulsion forces due to zeta potential, with these forces strengthening as surface charge increases [72]. The use of sonochemical synthesis further enhances stability by generating high-energy conditions that inhibit particle clustering [73], supporting the suitability of these F-AuNPs for biomedical applications.

In the present study, dynamic light scattering (DLS) analysis demonstrated that *T. decurrens* F-AuNPs averaged 98.5 ± 2.24 nm with a polydispersity index (PdI) of 0.349 ± 0.031, reflecting a relatively narrow size distribution, whereas *S. cinereum* F-AuNPs were larger (157.8 ± 5.55 nm) and exhibited a slightly broader PdI (0.390 ± 0.010). These outcomes are consistent with the recent literature on fucoidan-mediated gold nanoparticles, which often report size ranges between 100 and 160 nm and PdI values of 0.32–0.4, depending on source and synthesis method [74,75]. Such variability is largely attributable to species-specific structural characteristics of fucoidan, particularly differences in molecular weight, sulfation patterns, and branching, which directly influence nanoparticle nucleation and stabilization mechanisms. From a biomedical perspective, nanoparticles within the 100–200 nm size range and PdI below 0.4 are regarded as optimal for drug delivery and diagnostic applications, given their favorable cellular uptake, circulation properties, and reproducibility. Thus, our findings not only corroborate the premise that fucoidan structure governs nanoparticle dimensions and uniformity but also underscore that *T. decurrens* F-AuNPs, with their compact size and acceptable homogeneity, are particularly promising candidates for downstream biomedical applications.

The DTG curve of *T. decurrens* fucoidan displayed a sharp peak between 250 and 350 °C, indicating a rapid and uniform thermal decomposition, likely due to a more regular molecular structure or reduced branching. A major weight loss between 200 and 400 °C reflects the degradation of fucoidan chains, including cleavage of glycosidic bonds into smaller fragments [76,77]. In contrast, the broader decomposition range in *S. cinereum* suggests structural heterogeneity, such as variable sulfation patterns or linkage types, leading to staggered thermal breakdown [78]. This thermal behavior comparison provides additional evidence of compositional differences between the two fucoidan types.

A secondary weight loss phase from 600 to 750 °C, especially pronounced in *T. decurrens*, may correspond to the decomposition of carbonaceous residues or thermally stable polysaccharide remnants [79]. As gold remains stable at these temperatures, the remaining non-volatile residue confirms the presence of the inorganic nanoparticle core [80].

The antioxidant potential of F-AuNPs synthesized from *T. decurrens* and *S. cinereum* was evaluated using both the DPPH free radical scavenging assay and the ferric-reducing antioxidant power (FRAP) assay. The DPPH assay demonstrated that *S. cinereum*-derived F-AuNPs exhibited the highest free radical scavenging activity, achieving up to 80.49 ± 0.19% inhibition at 31.25 µg mL^−1^, compared to 78.11 ± 0.31% for T. decurrens-derived F-AuNPs. Consistent with these findings, Hasan et al. [81] reported that gold nanoparticles biosynthesized with Turbinaria decurrens extract displayed markedly stronger, dose-dependent DPPH and ABTS scavenging than the parent extract, confirming that nano-formulation amplifies the antioxidant power of fucoidan-rich seaweeds. Furthermore, the Trolox equivalent antioxidant capacity was higher in *S. cinereum* F-AuNPs (4.48 ± 0.42 µg TE/mg) than in *T. decurrens* F-AuNPs (3.44 ± 0.11 µg TE mg^−1^). Despite *T. decurrens* having higher sulfation, the superior antioxidant activity in *S. cinereum* F-AuNPs suggests the influence of other structural components, such as phenolics or lower-molecular-weight fractions. These findings suggest that *S. cinereum* F-AuNPs have superior radical-scavenging efficacy, making them strong candidates for inclusion in pharmaceutical and cosmetic formulations requiring potent antioxidant properties. Importantly, the crude fucoidan extracts themselves also displayed moderate DPPH radical scavenging activity. *T. decurrens* fucoidan achieved up to 73.09 ± 0.23% inhibition, while *S. cinereum* fucoidan reached 71.36 ± 0.21% at 31.25 µg mL^−1^. A comparable trend was observed by Nurhidayati et al., (2022) [82] who recorded notable DPPH-scavenging capacity for crude fucoidan isolated from *S. cinereum*, underscoring the inherent radical-quenching ability of native brown-algal polysaccharides.

Although these values are slightly lower than those of their nanoparticle counterparts, they confirm that the native polysaccharides possess inherent antioxidant activity, likely due to their sulfate content and phenolic constituents.

Conversely, the FRAP assay results indicated that *T. decurrens* F-AuNPs exhibited a greater ferric-reducing power, with a Trolox-equivalent capacity of 9.21 ± 0.30 µg TE mg^−1^, compared to 8.42 ± 0.27 µg TE mg^−1^ for *S. cinereum* F-AuNPs. These results suggest that *T. decurrens* F-AuNPs possess a more robust electron-donating capacity, which is critical in redox regulation and related biomedical applications. The crude fucoidan extracts also showed notable ferric-reducing activity, although to a lesser extent. At 400 µg mL^−1^, T. decurrens fucoidan reached 1.11 ± 0.01 absorbance units, slightly higher than *S. cinereum* at 1.09 ± 0.12. This suggests that even without nanoparticle conjugation, fucoidans from both seaweeds are capable of participating in redox reactions, albeit with reduced efficacy.

The enhanced antioxidant capacity of the F-AuNPs over their native fucoidan forms may be attributed to synergistic effects between the gold core and surface-bound fucoidan molecules, which could increase surface reactivity and radical-quenching potential. Moreover, structural variations in fucoidan—such as sulfation degree, molecular weight, and uronic acid content—may further influence their antioxidant efficacy and the stability of the functionalized nanoparticles. The literature supports the notion that fucoidan can improve nanoparticle performance by enhancing dispersion, reactivity, and biocompatibility in biological systems.

Research by Barakat et al. (2022) [18] demonstrated that ulvan, another sulfated polysaccharide from *Ulva fasciata*, contributed to strong antioxidant activity in synthesized nanoparticles. Similarly, fucoidan extracted from *Sargassum* and *Turbinaria* species may play a crucial role in the antioxidant performance of their respective F-AuNPs by providing functional groups that facilitate electron donation and free radical neutralization. Previous studies have reported that F-NPs exhibit enhanced DPPH scavenging and FRAP activity due to their polysaccharide-mediated surface modifications [67].

These findings suggest that fucoidan could be a key factor in modulating the antioxidant capacity of *T. decurrens* and *S. cinereum* F-AuNPs. Further studies on the structural characterization of fucoidan in these species and its specific role in nanoparticle synthesis could provide deeper insights into optimizing their antioxidant potential for biomedical and pharmaceutical applications.

Regarding the cytotoxicity assessment, the fucoidan-mediated gold nanoparticles (F-AuNPs) synthesized from *Turbinaria decurrens* and *Sargassum cinereum* demonstrated selective cytotoxic effects. Specifically, the MTT assay on HepG2 cells revealed IC_50_ values of 449.5 µg mL^−1^ for *T. decurrens* and 377.6 µg mL^−1^ for *S. cinereum*, indicating moderate cytotoxicity against hepatocellular carcinoma cells (Figure 14A,B). In contrast, both formulations exhibited substantially higher IC_50_ values in THP1 cells, suggesting low cytotoxicity within the tested range and a potentially favorable safety profile for hematologic cell types. *Additionally*, SRB assay results for normal murine hepatic cells (BNL) demonstrated minimal toxicity, with cell viability exceeding 85% across all tested concentrations and estimated IC_50_ values greater than 1000 µg mL^−1^ for *T. decurrens* and approximately 800 µg mL^−1^ for *S. cinereum*. According to established cytotoxicity criteria, nanoparticles maintaining cell viability above 70% are generally considered biocompatible [83]. These findings indicate that the F-AuNPs possess selective cytotoxic activity toward cancerous cells while sparing non-malignant ones, making them suitable candidates for further evaluation as anticancer agents or diagnostic nanoprobes [68]. Consistently, the calculated selectivity indices (SIs) obtained from the IC_50_ ratio BNL/HepG_2_ were greater than 2 for both formulations. This meets the widely accepted minimum threshold for selective anticancer agents [84,85], and it is comparable to SI values reported for other biogenic AuNP systems [86]. These figures delineate a favorable therapeutic window and warrant in vivo validation, alongside further optimization of particle size, surface charge, or fucoidan sulfation, to enhance tumor specificity. Mechanistically, gold nanoparticles smaller than 10 nm have been reported to efficiently penetrate tumor cell membranes and accumulate intracellularly, inducing cytotoxic effects through oxidative stress and mitochondrial dysfunction [87,88]. It is plausible that the cytotoxicity observed in HepG2 cells is mediated through such size-dependent internalization mechanisms, although further mechanistic studies are warranted.

Previous studies have also demonstrated the effectiveness of AuNPs in the colon, [89] breast, [90], cholangiocarcinoma, [91] cervical, and ovarian [92] cancer cell lines, and our present results are constant with the previous studies. These studies indicate that AuNPs can inhibit cancer cell proliferation, regardless of their synthesis method (chemical, Phyto, or microbial) [93]. Experimental findings demonstrated strong anticancer activity of AuNPs against the HeLa cell line [94,95]. Additionally, research by Hamed and colleagues confirmed the anticancer potential of AuNPs against carcinoma cell lines [96]. The antioxidant and cytotoxic effects of F-AuNPs are largely attributed to the bioactive functionalities of fucoidan on the nanoparticle surface. Mechanistically, fucoidan contains abundant hydroxyl and sulfate groups that donate electrons or hydrogen atoms to neutralize reactive oxygen species (ROS), thereby inhibiting oxidative stress—a key pathway in both cell damage and cancer progression. The surface-modified gold nanoparticles act synergistically, with fucoidan enhancing radical scavenging via increased surface reactivity and colloidal stability. In terms of cytotoxicity, F-AuNPs may enter tumor cells through endocytosis and, once internalized, induce apoptosis by generating ROS, disrupting mitochondrial membrane potential, and triggering caspase activation [97].

The molecular-docking results provide mechanistic insights into the anticancer potential of fucoidan-functionalized gold nanoparticles (F-AuNPs). Notably, F-AuNPs demonstrated moderate binding affinities to several key proteins implicated in cancer progression and inflammation, supporting their multifunctional bioactivity.

The observed interaction with arachidonate 5-lipoxygenase (ALOX5, −4.07 kcal mol^−1^) aligns with prior findings that link ALOX5 inhibition to reduced inflammation-driven carcinogenesis. ALOX5 is a critical enzyme in leukotriene biosynthesis, and its inhibition can impair pathways involved in ferroptosis and oxidative stress-mediated cell death [98].

The binding to Cyclooxygenase-2 (COX-2, −7.1 kcal mol^−1^) further supports the anti-inflammatory and anticancer profile of F-AuNPs. COX-2 is often upregulated in tumors and plays a central role in PGE2-mediated pathways that promote angiogenesis and immune evasion [99]. Prior studies have shown that gold nanoparticles functionalized with marine polysaccharides downregulate COX-2 expression, thereby limiting metastasis and tumor progression, consistent with our findings.

Telomerase reverse transcriptase (TERT, −5.4 kcal mol^−1^) and thymidylate synthase (TYMS, −4.06 kcal mol^−1^) are both involved in sustaining the uncontrolled proliferation characteristic of cancer cells. While the moderate binding energies suggest auxiliary roles rather than primary inhibition, these interactions reinforce the potential of F-AuNPs to interfere with telomere maintenance and nucleotide synthesis—two processes central to tumor cell immortality and chemoresistance. Similar binding trends to TERT and TYMS have been reported for fucoidan-derived nanocarriers, supporting their role in multi-targeted therapy approaches [100]. Cyclooxygenase-2 (COX-2) and telomerase reverse-transcriptase (TERT) were prioritized because both are well-validated drivers of hepatocellular carcinoma: COX-2 is over-expressed in HepG2 and other HCC cell lines, where its inhibition suppresses proliferation, angiogenesis and immune evasion [101,102], while activating TERT-promoter mutations and high telomerase activity occur in ≈ 60% of HCC tumors and in HepG2 cells, sustaining replicative immortality and poor prognosis [103,104].

A notable finding was the relatively stronger interaction with the histamine H3 receptor (H3R, −5.07 kcal mol^−1^), a less conventional but emerging target in hormone-responsive cancers like prostate cancer. The literature highlights H3R’s role in modulating androgen receptor pathways and promoting resistance to apoptosis. Our docking results suggest F-AuNPs may interfere with this axis, potentially disrupting survival signaling—a mechanism previously suggested for fucoidan-based therapies in hormone-dependent tumors [105].

In contrast, the lower binding energy to Cyclin-Dependent Kinase 11 (CDK11, –1.99 kcal mol^−1^) suggests limited interaction at the active site, though this does not preclude indirect modulation. Given CDK11′s role in transcription and cell cycle control, future work might explore whether F-AuNPs affect downstream regulators or expression levels [106].

In sum, these docking outcomes align with the observed cytotoxic effects against HepG2 cells and the low toxicity to THP1 cells, highlighting a promising therapeutic window. The multi-target binding pattern of F-AuNPs reflects a polypharmacological potential—simultaneously affecting inflammation, proliferation, and cell survival—consistent with previous studies on fucoidan-mediated nanoparticle bioactivity.

## 5. Study Limitations and Future Directions

Despite the promising in vitro anticancer activity of F-AuNPs, several limitations should be acknowledged. First, no in vivo efficacy or pharmacokinetic (PK)/biodistribution experiments were performed. Free fucoidan exhibits notoriously low oral bioavailability because its high molecular weight and dense sulfate groups hinder intestinal uptake, and it is rapidly cleared when administered intravenously [107,108]. Second, although polysaccharide coatings such as fucoidan can prolong nanoparticle circulation and double tumor accumulation in mice, these benefits remain hypothetical for the present system because we did not quantify plasma Au, tissue Au, or fucoidan levels after systemic administration [109]. Future investigations will include (i) in vivo antitumor testing of F-AuNPs in a HepG2 xenograft mouse model; (ii) full pharmacokinetic profiling C max, AUC, and t_½_ for both free fucoidan and F-AuNPs, quantified by LC–MS/MS (fucoidan-derived oligosaccharides) and ICP-MS (Au); and (iii) mechanism-specific gene/protein expression. These studies are designed to verify whether the in vitro superiority of F-AuNPs is maintained in vivo and to define their therapeutic window with translational precision.

## 6. Conclusions

This study demonstrates that fucoidan-functionalized gold nanoparticles (F-AuNPs) synthesized from *Sargassum cinereum* and *Turbinaria decurrens* exhibit promising antioxidant and anticancer properties. The successful synthesis and characterization of F-AuNPs were confirmed through UV-Vis spectroscopy, FTIR, XRD, TEM, and zeta potential analysis. In vitro assays revealed that F-AuNPs significantly enhanced cytotoxicity against HepG2 cells while maintaining biocompatibility with normal BNL cells. Additionally, molecular-docking analyses provided mechanistic insights into their potential interactions with key cancer-related proteins, including COX-2 and TERT, further supporting their anticancer potential. Despite these promising findings, further in vivo studies are required to assess the pharmacokinetics, biodistribution, and long-term toxicity of F-AuNPs. Future research should also explore targeted drug delivery strategies, such as surface modifications for selective tumor targeting, to enhance therapeutic efficacy. By integrating nanotechnology with marine bioactive compounds, this study provides a foundation for the development of novel, biocompatible nanomedicines for cancer treatment. The present in vitro findings position fucoidan-coated AuNPs as a promising platform; however, rigorous in vivo pharmacokinetic and efficacy studies comparing free versus nanoparticle-conjugated fucoidan are essential next steps to confirm therapeutic superiority.

## Figures and Tables

**Table 1:** The DPPH assay of fucoidan–gold nanoparticles (F-AuNPs) for two brown seaweeds.

Inhibition (%)					
Concentrations (µg mL^−1^)	Trolox	*S. cinereum* Fucoidan	*S. cinereum* F-AuNPs	*T. decurrens* Fucoidan	*T. decurrens* F-AuNPs
**3.90**	11.73 ± 0.21	7.92 ± 0.10	10.82 ± 0.11	9.15 ± 0.25	12.05 ± 0.41
**7.81**	25.92 ± 0.39	17.51 ± 0.05	21.61 ± 0.08	13.17 ± 0.14	24.07 ± 0.36
**15.60**	47.15 ± 0.42	37.13 ± 0.08	43.23 ± 0.13	35.16 ± 0.12	39.06 ± 0.12
**25.00**	68.64 ± 0.15	59.17 ± 0.14	66.19 ± 0.09	59.35 ± 0.26	62.49 ± 0.31
**31.25**	83.80 ± 0.18	71.36 ± 0.21	80.49 ± 0.19	73.09 ± 0.23	78.11 ± 0.31

**Table 2:** FRAP assay of fucoidan–gold nanoparticles (F-AuNPs) for two brown seaweeds.

Inhibition (%)					
Concentrations (µg mL^−1^)	Trolox	*S. cinereum* Fucoidan	*S. cinereum* F-AuNPs	*T. decurrens* Fucoidan	*T. decurrens* F-AuNPs
**25**	0.09 ± 0.01	0.02 ± 0.04	0.07 ± 0.02	0.04 ± 0.15	0.07 ± 0.00
**50**	0.15 ± 0.02	0.11 ± 0.12	0.14 ± 0.03	0.09 ± 0.04	0.14 ± 0.01
**100**	0.34 ± 0.06	0.21 ± 0.13	0.28 ± 0.04	0.16 ± 0.09	0.28 ± 0.07
**200**	0.65 ± 0.00	0.27 ± 0.06	0.56 ± 0.03	0.32 ± 0.21	0.57 ± 0.02
**400**	1.40 ± 0.10	1.09 ± 0.12	1.12 ± 0.18	1.11 ± 0.01	1.14 ± 0.19

**Table 3:** Molecular-docking results in terms of the binding energy of F-Au NAPs with protein receptors.

No.	Receptors	Nanoparticles	Binding Affinity (Kcal mol^−1^)
1	Arachidonate 5-lipoxygenase	F-Au NAPs	−4.07
2	Cyclooxygenase-2	F-Au NAPs	−7.1
3	Telomerase reverse transcriptase	F-Au NAPs	−5.4
4	Thymidylate synthase	F-Au NAPs	−4.06
5	Histamine H3 receptor	F-Au NAPs	−5.07
6	Cyclin-Dependent Kinases	F-Au NAPs	−1.99

## Data Availability

Data supporting these results are available upon reasonable request from the corresponding author.

## Supplementary Materials

The following supporting information can be downloaded at: https://www.mdpi.com/article/10.3390/pharmaceutics17070826/s1, Figure S1: DLS profiles replicates of F-Au-NPs from Turbinaria decurrens; Figure S2: DLS profiles replicates of F-Au-NPs for Sargassum cinereum.
