# Supplementary material for: Fucoidan-Based Gold Nanoparticles: Antioxidant and Anticancer Potential from Turbinaria decurrens and Sargassum cinereum
Source: Pharmaceutics. 2025 Jun 25;17(7):826. doi: 10.3390/pharmaceutics17070826 (PMC12299627; doi:10.3390/pharmaceutics17070826)
Supplement: Supplementary file 1 [file pharmaceutics-17-00826-s001.zip › pharmaceutics-3697780-supplementary.pdf]

(A) **Results**

			Size (d.nm):	% Intensity:	St Dev (d.n...
Z-Average (d.nm):	100.3	Peak 1:	142.1	98.1	84.86
PdI:	0.378	Peak 2:	4801	1.9	724.6
Intercept:	0.814	Peak 3:	0.000	0.0	0.000
Result quality :	Good				

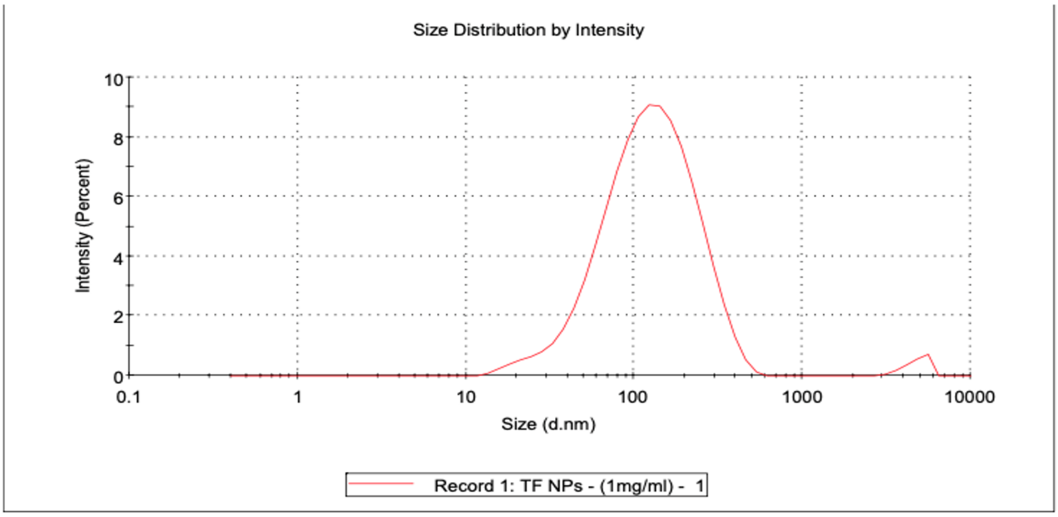

(B) **Results**

			Size (d.nm):	% Intensity:	St Dev (d.n...
Z-Average (d.nm):	99.21	Peak 1:	128.2	96.8	61.82
PdI:	0.317	Peak 2:	4530	3.2	863.9
Intercept:	0.813	Peak 3:	0.000	0.0	0.000
Result quality :	Good				

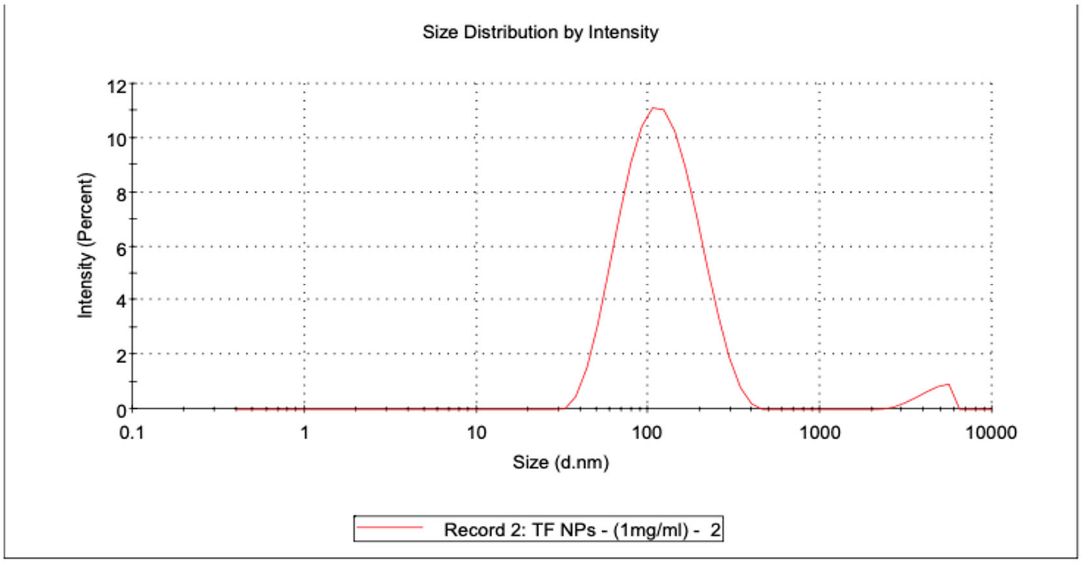

(C) **Results**

			Size (d.nm):	% Intensity:	St Dev (d.n...
Z-Average (d.nm):	95.99	Peak 1:	145.5	100.0	92.82
PdI:	0.352	Peak 2:	0.000	0.0	0.000
Intercept:	0.810	Peak 3:	0.000	0.0	0.000
Result quality :	Good				

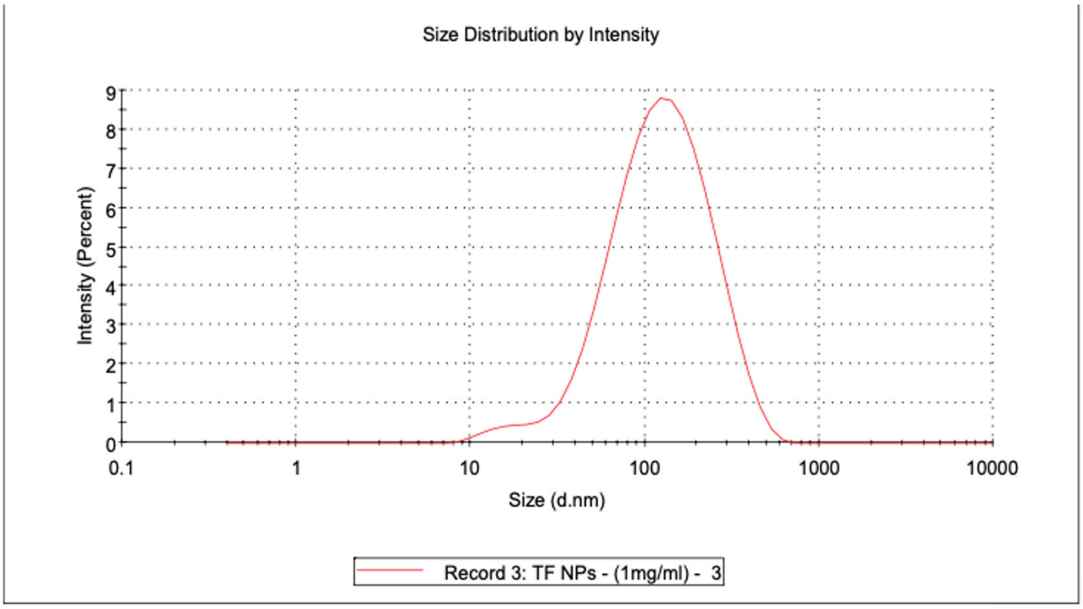

Figure S1. DLS profiles replicates of F-Au-NPs from *Turbinaria decurrens*.

(A) **Results**

			Size (d.nm):	% Intensity:	St Dev (d.n...
Z-Average (d.nm):	160.3	Peak 1:	220.1	93.2	135.2
PdI:	0.397	Peak 2:	4112	5.6	1045
Intercept:	0.831	Peak 3:	10.66	1.2	2.321
Result quality :	Good				

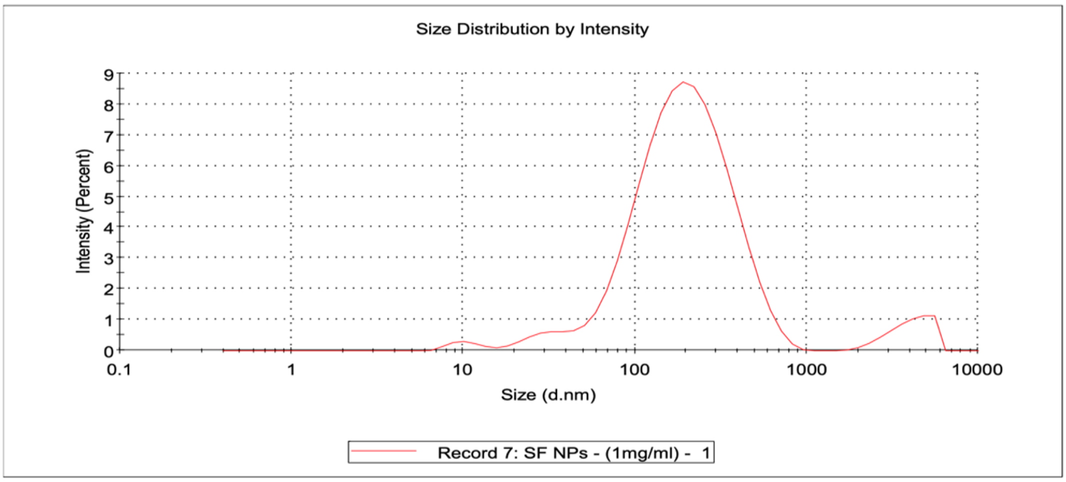

(B) **Results**

			Size (d.nm):	% Intensity:	St Dev (d.n...
Z-Average (d.nm):	161.6	Peak 1:	220.5	93.8	131.0
PdI:	0.379	Peak 2:	3865	6.2	1128
Intercept:	0.829	Peak 3:	0.000	0.0	0.000
Result quality :	Good				

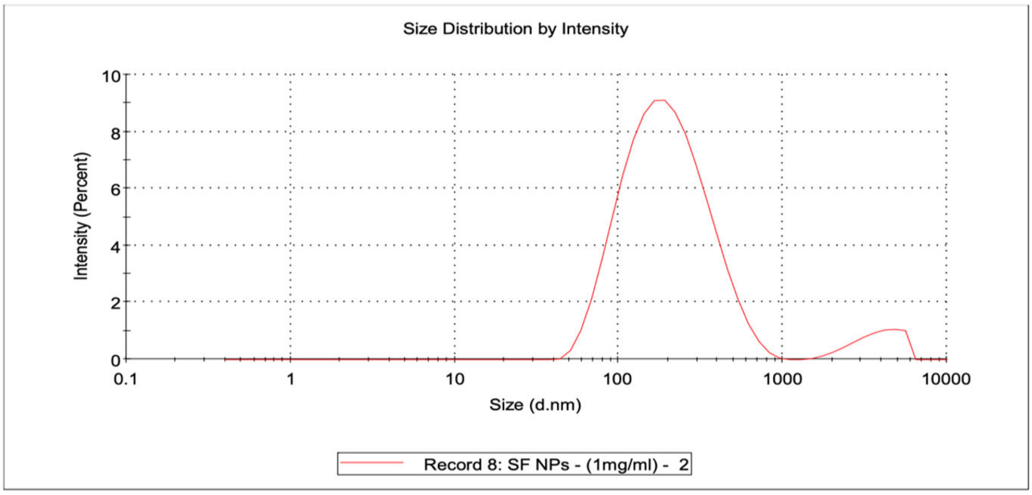

(C) **Results**

			Size (d.nm):	% Intensity:	St Dev (d.n...
Z-Average (d.nm):	151.4	Peak 1:	234.3	100.0	150.2
PdI:	0.394	Peak 2:	0.000	0.0	0.000
Intercept:	0.833	Peak 3:	0.000	0.0	0.000
Result quality :	Good				

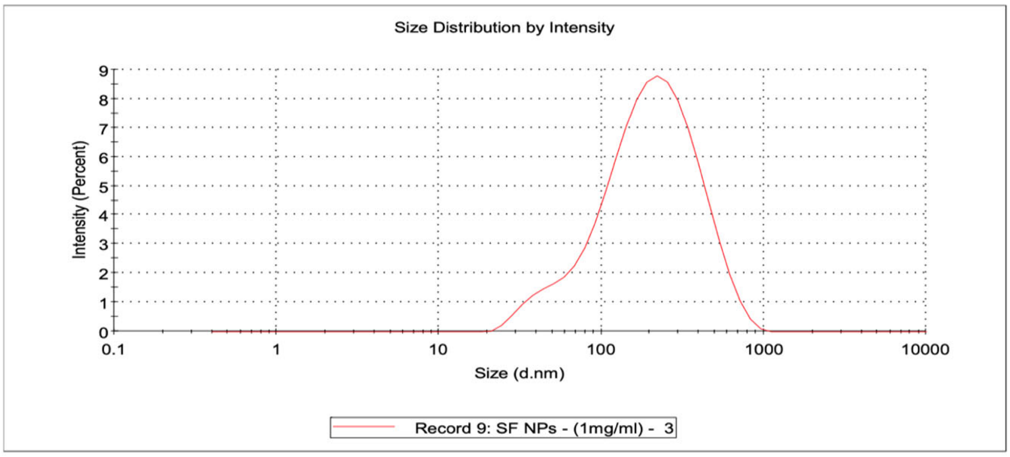

Figure S2. DLS profiles replicates of F-Au-NPs for *Sargassum cinereum*.
